# Population genetic structure of the maize weevil, *Sitophilus zeamais*, in southern Mexico

**DOI:** 10.1371/journal.pone.0264469

**Published:** 2023-04-12

**Authors:** Jennifer Baltzegar, Michael S. Jones, Martha Willcox, Janine M. Ramsey, Fred Gould

**Affiliations:** 1 Genetic Engineering and Society Center, North Carolina State University, Raleigh, NC, United States of America; 2 Graduate Program in Genetics, North Carolina State University, Raleigh, NC, United States of America; 3 International Maize and Wheat Improvement Center (CIMMYT), Texcoco, México; 4 Department of Agronomy, University of Wisconsin-Madison, Madison, WI, United States of America; 5 Instituto Nacional de Salud Pública/CRISP, Tapachula, Chiapas, México; 6 Department of Entomology and Plant Pathology, North Carolina State University, Raleigh, NC, United States of America; ICAR-Indian Institute of Pulses Research, INDIA

## Abstract

The maize weevil, *Sitophilus zeamais*, is a ubiquitous pest of maize and other cereal crops worldwide and remains a threat to food security in subsistence communities. Few population genetic studies have been conducted on the maize weevil, but those that exist have shown that there is very little genetic differentiation between geographically dispersed populations and that it is likely the species has experienced a recent range expansion within the last few hundred years. While the previous studies found little genetic structure, they relied primarily on mitochondrial and nuclear microsatellite markers for their analyses. It is possible that more fine-scaled population genetic structure exists due to local adaptation, the biological limits of natural species dispersal, and the isolated nature of subsistence farming communities. In contrast to previous studies, here, we utilized genome-wide single nucleotide polymorphism data to evaluate the genetic population structure of the maize weevil from the southern and coastal Mexican states of Oaxaca and Chiapas. We employed strict SNP filtering to manage large next generation sequencing lane effects and this study is the first to find fine-scale genetic population structure in the maize weevil. Here, we show that although there continues to be gene flow between populations of maize weevil, that fine-scale genetic structure exists. It is possible that this structure is shaped by local adaptation of the insects, the movement and trade of maize by humans in the region, geographic barriers to gene flow, or a combination of these factors.

## Introduction

The maize weevil, *Sitophilus zeamais*, is a ubiquitous pest of maize and other cereal crops worldwide. Although there are effective control solutions, these are not always available to subsistence farmers. Therefore, maize weevils remain a threat to food security in these communities [1]. Recent research to control the maize weevil has focused on developing maize varieties resistant to the insect, improving storage conditions, and identifying chemical compounds toxic to the species [e.g., 2–4]. Maize varieties that are resistant to damage tend to have higher levels of phenolic compounds, which increases the hardness of grain and provides mechanical resistance to the maize from the maize weevil [5]. However, subsistence farmers, especially in Mexico, often prefer to grow their own landraces, which are maize varieties adapted for the specific climate, taste, and texture preferences in the region and may not have been specifically selected to protect against storage pests [6]. Hermetic storage solutions can be very effective, but they can also be prohibitively expensive for subsistence farmers. Recently, advances in more affordable hermetic storage bags have been made and programs exist to give access to silos and these improved storage solutions. However, silos and other hermetic storage containers are still not available to all who need them. Insecticides are used frequently, but insecticide resistance has been reported in this species. And resistance to any new chemical control agents would be expected to develop with sustained use [7]. Therefore, despite having a myriad of control methods, maize weevils continue to cause grain loss, especially in subsistence communities [1].

Few population genetic studies have been conducted on the maize weevil and its sister species, the rice weevil, *S*. *oryzae* [8–12]. Those studies have shown that there is very little genetic differentiation between geographically dispersed populations [9, 11]. For maize weevil, eight nuclear microsatellite markers were previously used to assess 15 populations from 9 countries over 4 continents [9]. The conclusion of the study was that very little genetic structure exists globally. A second study used mitochondrial markers to identify population structure among 9 countries in West and Central Africa [11]. Again, the conclusion was that very little phylogeographic genetic structure exists in this species. Together, these results suggest the recent worldwide expansion of the maize weevil. Similarly, studies in the rice weevil and other pest of stored grain have also shown evidence for low levels of population structure [12, 13].

While a recent range expansion of the maize weevil has likely occurred within the last few hundred years, we speculate that more fine-scaled population genetic structure exists due to local adaptation, the biological limits of natural species dispersal, or the isolated nature of subsistence farming communities. Maize weevils are not strong fliers. The typical flight range for the species was roughly estimated to be one quarter mile [14]. While human mediated movement of grain via trade is generally understood to be a primary source of movement for stored pests, subsistence farmers are likely participating in grain trade at a reduced rate compared to larger commercial farmers, particularly at the international scale. Thus, if genetic differences are to be found among maize weevil populations, they are likely to be observed between subsistence communities in socially isolated regions.

Mexico was chosen as the study location as it is near the center of origin for maize and the maize weevil is the most common pest present in stored maize in Oaxaca and Chiapas. The region continues to have strong subsistence farming communities and each community grows landraces of maize specific to their region and preferences. Human mediated transport via the grain trade is likely the most common way that maize weevils migrate in this region. The specific amount of trade among subsistence households in Mexico is not well characterized because farmers do not prioritize this type of record keeping [6]. However, population genetic studies of maize grain in Oaxaca and Chiapas have shown that there are high amounts of gene flow at the same time that local population structure exists and it appears to correlate well with the social origin of the farmer [15, 16]. Seed is primarily saved from one year to the next on the same farm and when needed, is first traded among farmers within the same community. Only occasionally will farmers acquire seed from regional markets [16]. These trade practices have been associated with the farmers’ perception of risk. The least risky trades are with local people who are well known and the riskiest trades are with strangers from other communities [16]. While information about the grain trade can inform our hypotheses about maize weevil population structure, it cannot act as a direct proxy as maize weevils can easily move from one variety of maize to another.

Natural migration via flight is also a possible method of movement for the maize weevil. Oaxaca and Chiapas are separated by the Isthmus of Tehuantepec. The isthmus is the shortest land distance between the Pacific Ocean and the Gulf of Mexico and is a great source for wind energy [17]. Given that maize weevils typically fly short distances (~0.25 miles) and are not particularly strong fliers, the isthmus may provide a barrier to gene flow [14]. In addition, both states exhibit a wide range of elevations and climates, providing further opportunity for local adaptation to occur and population structure to be identified.

It is imperative to better understand the basic population dynamics underlying this species so that traditional control methods can better be applied. Furthermore, the information in this study will be vital for informing the socio-ecological context in which maize weevil exists, allowing researchers who might develop emerging technologies for maize weevil control to identify relevant stakeholders from which to seek approval [18].

Mitochondrial and nuclear microsatellite markers are classic methods to assess the population genetic structure in species of interest [19]. They are especially good at identifying population structure that has existed for a significant period of time. However, the use of so few markers limits the statistical power that these studies have to identify recently developed genetic structure. In addition, mtDNA should not be used as the primary marker to study the recent evolutionary history in species that carry inherited symbionts because indirect selection on mtDNA can occur due to disequilibrium with other maternally inherited genes [20]. Maize weevils host two endosymbionts: *Sodalis pierantonius* and *Wolbachia* [9, 10, 21–23]. In these cases, the finding of no structure is not proof for the absence of structure, rather it is likely that these studies are underpowered or contain biases [19, 20]. It is prudent to utilize a more statistically powerful technique to identify possible genetic structure. Sets of single nucleotide polymorphisms (SNPs) provide genetic markers that have higher statistical power to answer these types of questions and are now used regularly to elucidate population structure. One individual bi-allelic SNP is less powerful than an individual microsatellite because of the fewer number of alleles sampled. But, when hundreds or thousands of SNPs are sampled at once, the combined statistical power is higher than the few dozen microsatellite loci that may be available for a species [24]. Maize weevil has only 8 polymorphic and amplifiable microsatellite loci identified [9].

Historically, it was challenging to generate a large number of informative SNPs for a non-model organism. However, reduced genome representation sequencing methods allow researchers to sample genome-wide SNPs without the need for *a priori* knowledge of the genome. Reduced representation methods have given researchers a greater ability to characterize the genetic population structure of important, but non-model organisms. Here we use double-digest restriction-site associated sequencing (ddRadSeq) to identify informative SNPs and assess the genetic population structure of the maize weevil collected from subsistence communities in southern Mexico.

## Materials and methods

### Field collections

**Fig 1:**
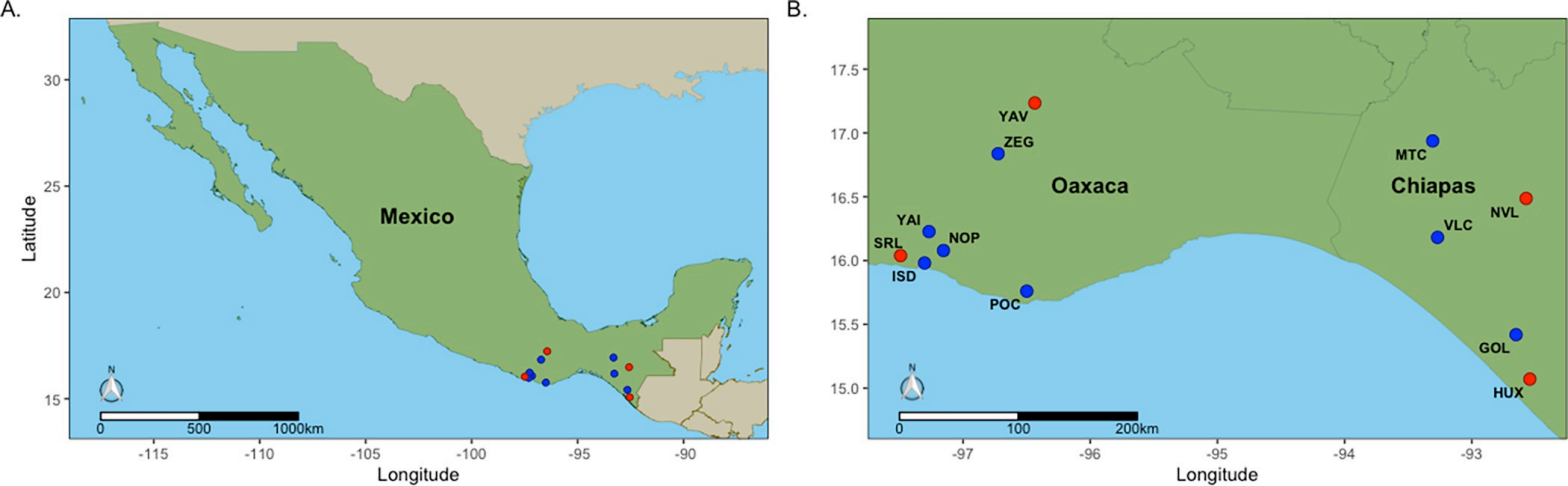
Map of sampling locations. Communities marked with red dots were sequenced in Lane 1, samples marked with blue dots were sequenced in Lane 2. Full community names are listed in Table 1. A) A map of Mexico showing relative location of sampled communities located in the southern and coastal region of Mexico. B) A zoomed in view centered on Oaxaca and Chiapas and the location of the communities where maize weevils were collected.

From 2 August to 18 August 2016, maize weevils were collected from a total of 61 small to medium sized farms within rural communities in southern Mexico. Weevils collected from each community were treated as one population in subsequent analyses. The sample locations were chosen based on previously established relationships with community members. Members of each residence verbally consented to specimen collection, but the need for review was waived by the ethics committee. We sampled 7 communities in Oaxaca, Mexico and 5 communities in Chiapas, Mexico (Fig 1). In each community, we collected approximately 200 maize weevils from 3 to 7 individual residences. These samples were stored in 100% ethanol at ambient temperature during the duration of field collecting. Upon returning to the United States, samples were stored at -20˚C in the laboratory until processed. At each sampling location, we recorded latitude, longitude, and elevation using a Garmin GPS unit (Garmin International, Inc., Olathe, KS, USA) (Table 1). The sites in Oaxaca ranged in elevation from 23.5 m to 2,005 m and those in Chiapas ranged from 8.0 m to 1,142.5 m.

**Table 1:** Summary table of communities visited in Oaxaca and Chiapas.

State	Community	Label	Homes	Elevation	Latitude	Longitude
Oaxaca	Santa María Yavesía	YAV	6	1988.25	17.234986	-96.436065
	Santa Ana Zegache	ZEG	4	1482.94	16.837518	-96.724833
	Santos Reyes Nopala	NOP	7	466.57	16.078580	-97.154197
	San Isídro Campechero	ISD	7	105.53	15.980961	-97.302886
	Santiago Yaitepec	YAI	5	1801.40	16.226998	-97.268016
	Santa Rosa de Lima	SRL	3	24.27	16.039082	-97.491641
	San Pedro Pochutla	POC	6	145.58	15.760327	-96.499309
Chiapas	Huixtla	HUX	5	13.72	15.071078	-92.543628
	Golondrinas	GOL	4	745.00	15.419210	-92.651981
	Montecristo	MTC	4	329.31	16.937728	-93.307673
	Villa Corzo	VLC	5	585.65	16.181779	-93.269561
	Nuevo León	NVL	5	1106.10	16.486764	-92.572740

Table 1 includes the label for each community, which corresponds to Fig 1 labels, number of households visited, mean latitude and longitude for each community, and mean elevation (m) of households visited in each community.

### Genomic DNA isolations

Genomic DNA was isolated from the head and thorax of individual weevils following the DNeasy Qiagen Kit (Qiagen Inc., Germantown, MD, USA) manufacturer’s suggestions in our laboratory at North Carolina State University in Raleigh, NC. DNA from 24 weevils from each of four communities (SRL, YAV, HUX, and NVL) were isolated for the proof-of-concept sequencing run. DNA from 12 weevils from each of the 8 remaining communities were isolated for the second sequencing run. Homogenized weevil tissue samples were incubated overnight in the lysis buffer solution at 55˚C and on day two of the isolation a RNaseA (4mg/mL) treatment was performed before the recommended wash steps. Finally, samples were eluted into 300 μl of 70˚C dH_2_O and stored at -20˚C for short term storage or -80˚C for long term storage.

### ddRadSeq library preparation and sequencing

Genomic DNA isolated from individual weevils as described above was prepared for sequencing following the ddRadSeq protocol first described by Peterson *et al*. [25] and adapted to our laboratory as described by Fritz *et al*. [26]. Two separate libraries were prepared and sequenced on two different lanes of an Illumina HiSeq 2500 125 bp SE. The first library included 96 individual weevils, 24 each from the 4 most distant communities sampled: Santa Maria Yavesia, Santa Rosa de Lima, Huixtla, and Nuevo Leon. This sampling regime included the communities with the highest and lowest elevation from each state. This particular library was used to test the hypothesis that genetic differentiation could be found by genotyping SNPs. A second library, sequenced in the same manner as the first, was prepared after confirmation that genetic differentiation could be identified. This library included 12 individual weevils from each of the remaining 8 communities sampled, Santa Ana Zegache, Santos Reyes Nopala, San Isidro Campechero, Santiago Yaitepec, Pochutla, Golondrinas, Montecristo, and Villa Corzo. The communities in the second library represent intermediate distances and elevations as compared to the first library.

### Bioinformatic and statistical analyses

Data produced by each lane was first checked with FastQC, then reads were trimmed with trimmomatic to remove adapter and Illumina indices [27, 28]. The trimming was verified by FastQC and a multiQC report was generated to compare results from the different indices [27–29]. Data from both lanes were analyzed together in Stacks v. 1.48 following the *de novo* procedure described by Rochette and Catchen [30, 31]. The procedure is outlined here to specify certain parameter choices. Samples were first cleaned, demultiplexed, and truncated to 115 bp using the program *process_radtags*. The per sample coverage was checked. Then, *ustacks* was run on all samples (-M = 5, -m = 3). The 4 highest coverage individuals from each of the 12 populations were chosen to create the catalog, with the exception of sample zag.c1, which had much higher coverage than other samples. Using the catalog popmap created in the previous step, *cstacks* was implemented (-n = 5). Then, *sstacks* was run on all samples. To improve calls, *rxstacks* was used with the flags—prune_haplo and—conf_lim = 0.10. Then, *cstacks* and sstacks were run again using the improved catalog. Finally, genotype information for each individual was exported from Stacks in genepop format from the *populations* program. Using the *adegenet* package in R, these data were stringently filtered [32, 33]. Individuals with greater than 80% missing genotype calls were removed. Then, loci with greater than 35% of missing data were removed. Principal components were then computed and plotted with the same program. F statistics were calculated in *hierfstat* package in R [34].

## Results

### ddRadSeq

**Fig 2:**
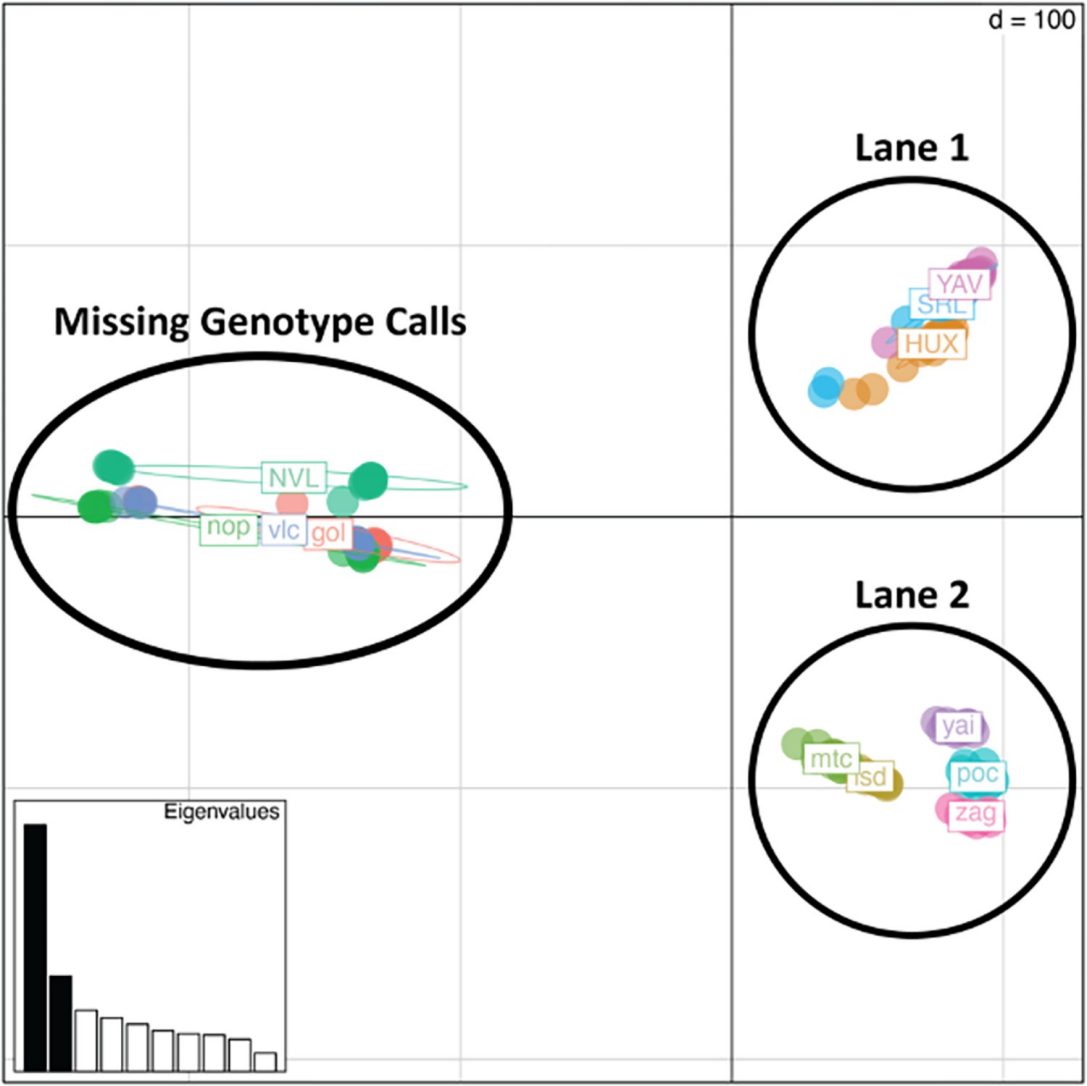
Principal components analysis of genetic variance illustrating the effect of sequencing lane and missing genotype calls for individuals in certain populations. Each population is color coded and labeled. Each effect group is circled and labeled. The four populations missing genotype calls were missing at least 80% of the genotypes output by populations in Stacks.

The first sequencing run resulted in 111,204,487 reads after trimming and filtering with *process_radtags*, with an average 1,158,380 reads per individual and a range of 288,190–3,079,538. The second sequencing library resulted in 186,219,498 trimmed and processed reads, with a range of 15,068–4,730,073 and an average 1,939,786 reads per individual. Although the initial quality check appeared similar in each sequencing run, there were clear sequencing lane differences upon inspection of the PCA plot produced after running *populations* with both lanes together in Stacks (Fig 2) therefore, stringent filtering criteria were applied. There were four populations, Santos Reyes Nopala (NOP), Golondrinas (GOL), Villa Corzo (VLC), and Nuevo Leon (NVL), where every individual had greater than 80% missing genotypes in the *populations* output, these were removed from further analysis. The populations with missing data were from both sequencing lanes. The mean number of reads for these populations following the *process_radtags* procedure was 1,680,432, while the mean number of reads for all populations was 1,549,083. Therefore, the reason for the high number of missing genotypes is likely due to specific parameter selection downstream of the *process_radtags* program. Following individual removal, a missing data threshold for individual SNPs was set at 35%, this resulted in the removal of 21,365 SNPs from the analysis. The final number of individuals included in the analysis was 132. The final number of SNPs included was 17,966.

### Statistical analyses

**Fig 3:**
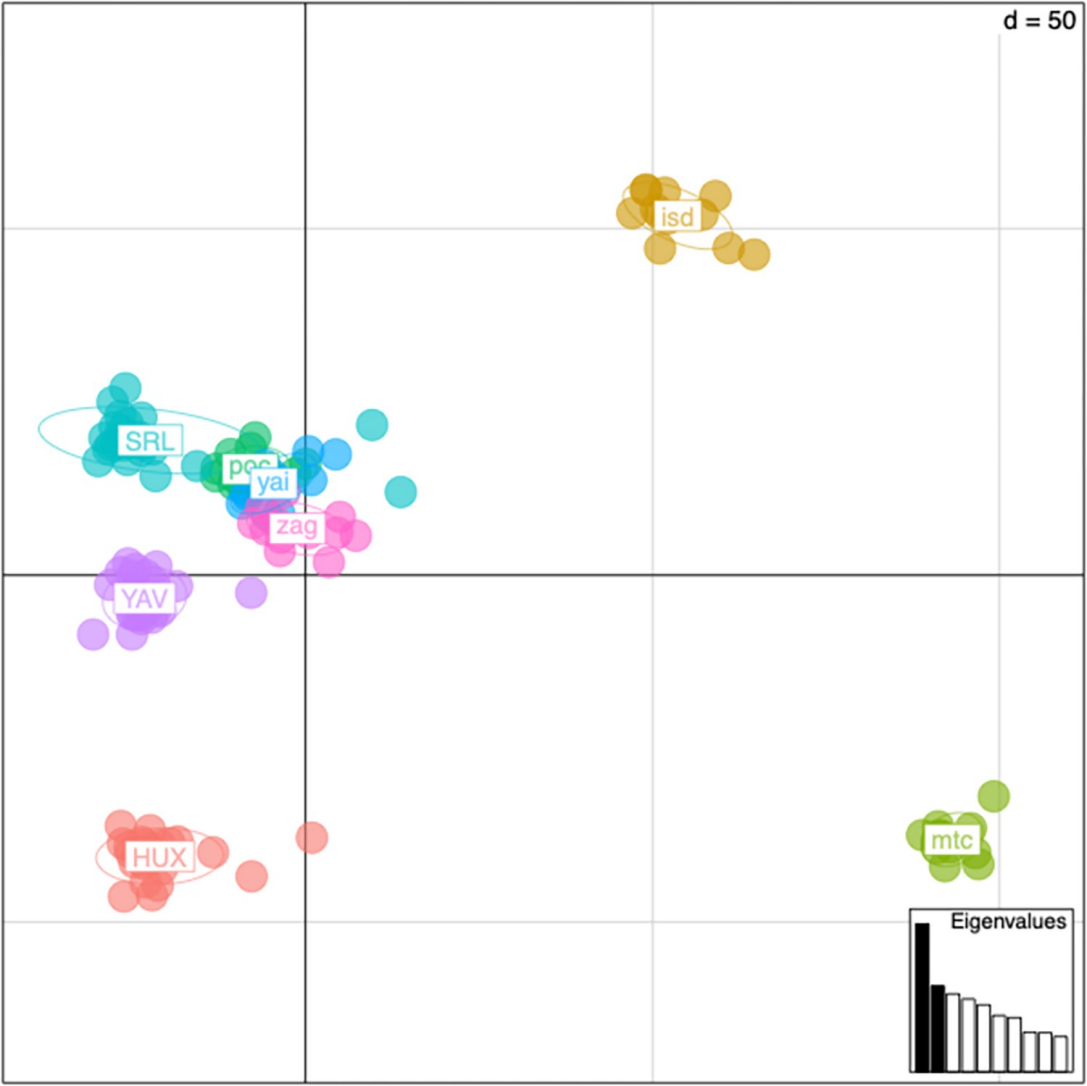
Principal components analysis of genetic variance in filtered data. Weevils from each community are color coded. Principal components 1 and 2 are plotted. Communities SRL, YAV, and HUX are from the first sequencing lane. The remaining populations are from the second sequencing lane. Principal component one is primarily explaining the difference between the two sequencing lanes.

**Fig 4:**
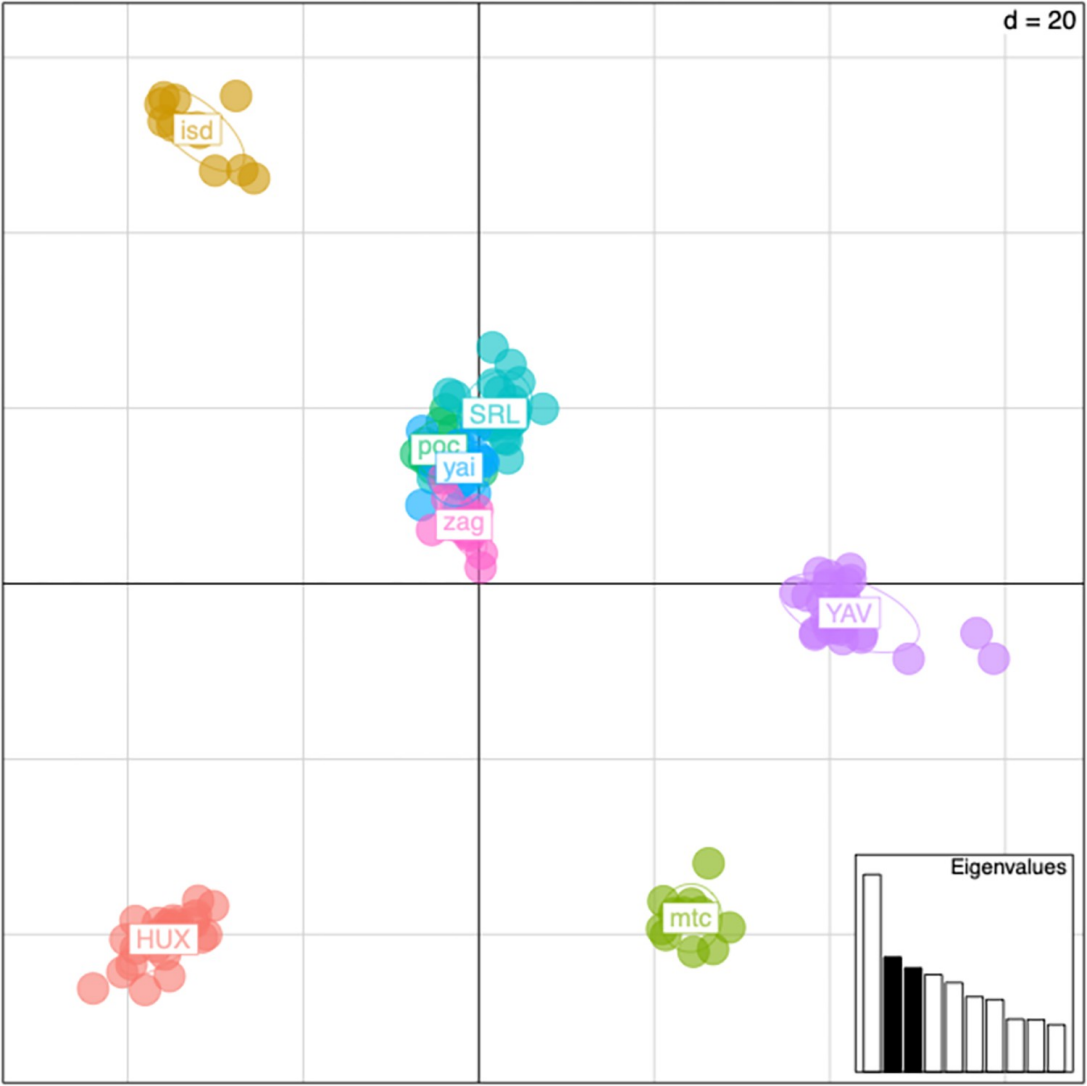
Principal components analysis of genetic variance in filtered data. Weevils from each community are color coded. Principal components 2 and 3 are plotted. Communities SRL, YAV, and HUX are from the first sequencing lane. The remaining populations are from the second sequencing lane.

Plotting principal components 1 and 2 for the filtered SNPs shows that the first principal component explains the difference between the two sequencing lanes (Fig 3). Plotting principal components 2 and 3 removes the lane effect and shows that weevils from each community group tightly together. There is a group of four communities in Oaxaca that share gene flow and cluster together, while the remaining communities are distinct from one another (Fig 4). In addition, principal component 3 can be explained by which state the community is located. The two communities on the bottom of the graph, Huixtla (HUX) and Montecristo (mtc), are in Chiapas, while all other communities are located in Oaxaca (Fig 4). Nei’s pairwise F_ST_ was calculated using the hierfstat package in R (Table 2). All population pairs have negative F_ST_ values, which should be interpreted as no difference between populations.

**Table 2:** Nei’s pairwise F_ST_.

	HUX	ISD	MTC	POC	SRL	YAI	YAV
**ISD**	-0.175						
**MTC**	-0.214	-0.237					
**POC**	-0.106	-0.198	-0.260				
**SRL**	-0.100	-0.167	-0.203	-0.099			
**YAI**	-0.113	-0.219	-0.269	-0.121	-0.113		
**YAV**	-0.093	-0.164	-0.197	-0.090	-0.091	-0.103	
**ZEG**	-0.111	-0.205	-0.256	-0.120	-0.108	-0.122	-0.099

Values calculated in the *hierfstat* package in R for the stringently filtered SNP data. Analysis includes 8 populations and 132 individuals.

## Discussion

Many studies have shown that low genetic structure is a common occurrence in pests of stored grain [12, 13], including in maize weevil [9, 11]. However, few of these studies have used markers with the power to identify recent and ongoing local adaptation [20, 35]. In this study, we utilized genome-wide SNPs to evaluate the genetic population structure of maize weevil, *S*. *zeamais*, from the southern and coastal Mexican states of Oaxaca and Chiapas. While other studies have attempted to identify genetic structure of this species at a larger scale, our smaller geographic scale study is the first to find fine-scale genetic population structure in the maize weevil.

In our data, we observe both negative pairwise F_ST_ estimates and apparent population genetic structure through principal components analysis. Negative F_ST_ values can generally be interpreted as zero and indicate that there are similar allele frequencies between populations [36]. This can occur if populations are artificially selected along a continuous gradient or if selection is acting on only a subset of loci sampled. F_ST_ estimates take into account information from all loci equally. The effect is that these estimates are biased toward older evolutionary events and that recent selection events or selection events on specific loci are dampened. PCA reduces the dimensionality of the dataset while maximizing variance. The effect of this approach gives more weight to the recently diverged loci. This allows PCA to identify local adaptation even when little to no population structure can be identified through F_ST_ analysis [37].

While there remains a high amount of gene flow between the sampled communities, the principal components analysis was able to identify some genetic differentiation based on geography. Primarily, there was a clear distinction between communities in Oaxaca and Chiapas. Four of the communities in Oaxaca (SRL, POC, YAI, ZEG) clustered very tightly and share a high amount of genetic similarity, but maize weevils from San Isidro Campecheros (ISD) and Santa Maria Yavesia (YAV) in Oaxaca, each formed their own distinct clusters. This is an interesting result as ISD is geographically close to the four communities that clustered together and is actually closer to SRL and YAI than POC and ZEG are. The exact reasons underlying the genetic structure are unknown, but we observed that the roads between certain communities in Oaxaca were easier to traverse and the travel time between sites was subsequently reduced. ISD was harder to reach via car and was more socially isolated than its neighboring communities. Likewise, YAV, the other genetically distinct community in Oaxaca was the most socially and geographically isolated community sampled in that state. The communities sampled in Chiapas were also much more isolated than most of those sampled in Oaxaca. These patterns resemble those found in the genetic population structure of maize from this region. While there is a high amount of gene flow among populations, fine-scale structure exists and is correlated with the social structure of farmers [15, 16]. It appears that maize weevil population structure may follow similar patterns.

The fact that the communities are more connected in Oaxaca than in Chiapas may help to facilitate grain trade between communities. These social interactions may be leading to more gene flow between maize weevil populations of the more connected Oaxacan communities versus the more isolated communities in each state. An interesting future study could incorporate farmer trade patterns into the genetic analysis to further explore the potential effect of human interactions on the genetic structure of maize weevil. This could be especially informative if supply chain nodes and trade endpoints were included in the sampling regime. If the effect is found to be significant, trade practices could also inform pest management strategies.

An alternative hypothesis to explain the difference between the populations found in Oaxaca and Chiapas is the existence of the Isthmus of Tehuantepec, an extremely windy area of Mexico. This region may form a natural barrier to weevil dispersal if migration is dominated by weevil flight rather than human-mediated grain trade. One or a combination of these factors may be shaping the genetic population structure of maize weevil in southern Mexico. Additional studies should seek to elucidate the underlying mechanisms contributing to the genetic structure observed in this study.

An important result presented here is the control of sequencing lane effects caused by combining independent sequencing lanes. The effects were controlled by stringent filtering of both individuals and loci. Removing under representative SNPs from the dataset greatly decreased the obvious lane effects. At the same time, a sufficient number of informative SNPs were retained to allow for clear relationships between populations to be observed. While it is preferable to design a ddRadSeq experiment where samples from all populations are randomized across all sequencing lanes, it is reassuring that with proper filtering and conservative SNP selection, this method is robust to identify population structure when randomization cannot be achieved.

This study is the first to characterize fine-scale population structure in the maize weevil. Previous research showed that the maize weevil has experienced a worldwide range expansion over the last several hundred years [9]. Here, we show that although there continues to be gene flow between populations of maize weevil, that fine-scale genetic structure exists. It is possible that this structure is shaped by the movement and trade of maize by humans in the region, by geographic barriers to gene flow, or a combination of factors.
